# Ecology and Prediction of Compensatory Growth: From Theory to Application in Forestry

**DOI:** 10.3389/fpls.2021.655417

**Published:** 2021-07-05

**Authors:** Chao Li, Hugh Barclay, Bernard Roitberg, Robert Lalonde

**Affiliations:** ^1^Canadian Wood Fiber Centre, Canadian Forest Service, Edmonton, AB, Canada; ^2^Pacific Forestry Centre, Canadian Forest Service, Victoria, BC, Canada; ^3^Department of Biological Sciences, Simon Fraser University, Burnaby, BC, Canada; ^4^Department of Biology, University of British Columbia, Okanagan, BC, Canada

**Keywords:** disturbance, overcompensation, partial mortality, plant response manipulation, productivity enhancement, scaling-up, state-dependent model, tree growth pattern

## Abstract

Compensatory growth has been observed in forests, and it also appears as a common phenomenon in biology. Though it sometimes takes different names, the essential meanings are the same, describing the accelerated growth of organisms when recovering from a period of unfavorable conditions such as tissue damage at the individual level and partial mortality at the population level. Diverse patterns of compensatory growth have been reported in the literature, ranging from under-, to compensation-induced-equality, and to over-compensation. In this review and synthesis, we provide examples of analogous compensatory growth from different fields, clarify different meanings of it, summarize its current understanding and modeling efforts, and argue that it is possible to develop a state-dependent model under the conceptual framework of compensatory growth, aimed at explaining and predicting diverse observations according to different disturbances and environmental conditions. When properly applied, compensatory growth can benefit different industries and human society in various forms.

## Introduction

The dynamics of any forest are likely to emerge as a function of a number of variables and processes, including but not limited to: natural perturbations (e.g., fires), species-specific responses to such perturbations, community structure, age structure, and access to limiting resources such as nitrogen (Harper, 1977; Davis et al., 2001). Given the complex nature of forests, it is not surprising that we may observe a very wide range of growth dynamics (e.g., Huang et al., 2009). As such, one of the great challenges is to develop forest management policies for optimizing fiber production across aging dynamics associated with widely divergent age and community structures. It is unlikely that any one policy will be optimal for all forest species or habitats.

We argue, however, that it is possible to develop best practice guidelines if one can construct the appropriate framework to elucidate the aforementioned variation in forest productivity within and among different plantings or natural communities. We further argue that such a framework is already well-developed for a number of non-forest biological systems and that we can avoid reinventing the wheel by incorporating the relevant concepts from life history theory and population ecology, as well as animal and plant sciences. Finally, while our focus is on silvicultural practices, our approach could also be applied to natural forests.

The approach that we advocate requires a two-step process (i) elucidating growth and reproductive dynamics for individual trees from the context of life history theory and (ii) scaling up from the individual organism to the (forest) community level. The former has received attention for a wide variety of organisms from both the plant and animal kingdoms as surveyed below (sections Compensatory Growth in Trees and Analogous Compensatory Growth in Other Plants and Animals), while the latter process is less well-developed.

In what follows, we will draw on a key concept, compensatory growth (CG). Here, we generalize our original definition from Li et al. (2020), to a change in growth rate, usually positive, in a forest stand following a disturbance that reduces biomass and/or individuals from the population.

The concept of CG is relatively new to the field of tree biology and forestry. However, it can provide a unique lens to explain diverse forest growth patterns after a partial mortality, and sometimes even controversial results from silviculture research. For instance, CG provided good explanation for the results from the Shawnigan Lake trial, in which gross volume in most precommercially thinned coastal Douglas-fir plots in British Columbia, Canada, exceeded that from control, 40 years after initial treatments (Li et al., 2018). A similar observation of such apparent overcompensation was also reported in a Balsam fir trial in New Brunswick, Canada (Pitt and Lanteigne, 2008).

Benefits from this enhanced productivity through thinning are apparent; not only yielding more wood for human society but also through many ecosystem services that are positively correlated to forest productivity such as carbon storage, and thus contribute to natural climate solution. Due to the uniqueness of the Shawnigan Lake dataset (multiple repeated measurements over 40 years), it may not be realistic to obtain similar datasets anytime soon. Li et al. (2020) explored the overcompensation from the Shawnigan Lake trials to other tree species, site conditions, and geographical regions. In the current review, we extend the above outcomes to address the following questions for encouraging CG research in forestry:

- What is compensatory growth and what forms does it take?
- How common is CG in biology?
- What is the contemporary understanding of CG from different fields?
- How is CG predicted in different fields?
- What are the analogs and differences in CG research?
- How can forestry and other industries benefit from CG research?

### What Is Compensatory Growth?

One of the key concepts in our discussion is that of CG, as defined above, which is a component of the total Density-Dependent (D-D) response to disturbances such as fire, insect attack, death of senescent trees, precommercial thinning, etc. After a disturbance, tree and stand biomass may continue increasing for some time beyond those of the undisturbed forest using the CG model, if the limiting factor for a given tree population is light and the population numbers are low enough that crown closure has not yet occurred.

The phenomenon of CG is not new in animal and plant sciences and can be traced back to about a century ago (e.g., Osborne and Mendel, 1915; Eaton, 1927). It can take place at the individual tree level via the increased allocation of photosynthate to somatic growth or reproduction, or at the stand level via changes in mortality or reproduction. For individual trees, the somatic growth response can be the increased rate of diameter and height growth (Bose et al., 2018), the increased rate of production of leaves (Rea and Massicotte, 2010), coppicing (Guillet and Bergström, 2006), increased root production (Baskin, 2013), etc. For reproductive response, it may consist of increased rates of production of seeds (i.e., fecundity), increased rates of germination of seeds (i.e., fertility) or faster growth of suppressed seedlings, etc. (Chambers, 1995; Jutila and Grace, 2002; Martini and Santos, 2007; Ascoli et al., 2019).

In the case of trees, disturbances may be due to arthropod attack, disease, fire, lightning strikes, floods, drought, and blowdown (e.g., Li, 2000; Schelhaas et al., 2003; Keane et al., 2004; Seidl et al., 2011; Gustafson and Sturtevant, 2013; Kretchun et al., 2014; Navarro et al., 2018; Díaz-Yáñez et al., 2019; Lavoie et al., 2019). Death of large trees may also occur via senescence, or from anthropogenic disturbances such as harvesting, thinning, or pruning (Pitt and Lanteigne, 2008; Hevia et al., 2016; Bose et al., 2018; Li et al., 2018). Each of these causes leaves a characteristic signature consisting of patterns of trees remaining following the disturbance, and so each will have somewhat different implications for the kind of growth response to be expected. Changes in growth in trees is likely a result of increased access of the remaining trees to resources, such as light, water, nutrients, and growing space following disturbances such as fires, or to deaths due to attacks by insects or disease, or to thinning.

An example of the outcome of CG is illustrated by the negative 3/2 power rule for self-thinning in the growth of a forest following crown closure (Yoda et al., 1963; Harper, 1977), which is a phenomenological description of the action of reduced fertility and increased mortality as a forest ages. Following a disturbance, an increase in growth rate is commonly observed until the forest recovers to the state of an undisturbed forest with crown closure, rejoining the status of the forest described by the −3/2 power curve. This accelerated growth during recovery constitutes CG.

The concept of CG seems obvious and simple: trees alter their growth rates when conditions change, yet a survey across or even within species will show that such responses can vary dramatically. A good example can be found in the response of various coastal Douglas-fir stands following different precommercial thinning (PCT) regimes (see the section Compensatory Growth of Costal Douglas-Fir). This illustrates the need for a general theory that can be applied to specific cases. Crucial to this theory is that there is a mechanistic underpinning and experimental validation.

CG is a special case of growth response and needs to be triggered by a period of unfavorable conditions (Li et al., 2020). It can provide a suitable framework to explain many observed growth response patterns and serve as a possible mechanistic contribution to the resilience of forest ecosystems. With enough time, CG could generate a state wherein stand volume could exceed that from undisturbed stands and thus stand productivity is enhanced to favor the forest sector as a whole. Consequently, identification of the conditions that promote stand productivity would benefit forestry practice, and serve the ultimate goal of silvicultural treatments (Li et al., 2020).

### Diverse Forms of Compensatory Growth

**Figure 1:**
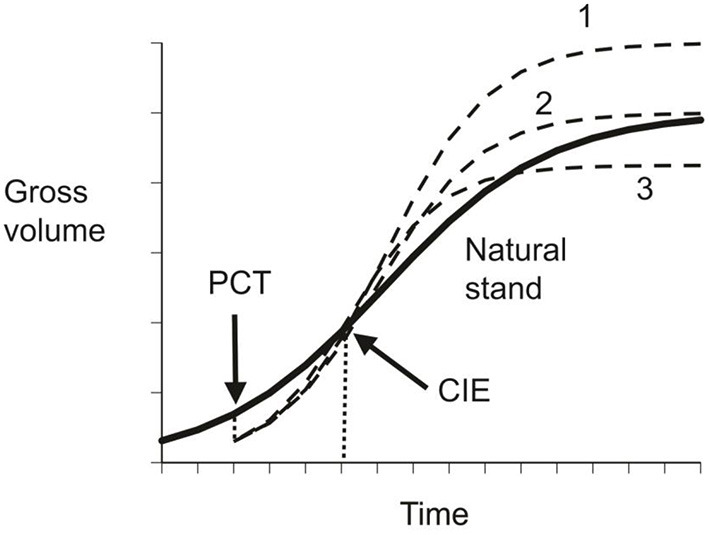
Schematic illustration of compensatory growth. The solid line represents gross volume in natural stands, the dished lines indicates possible compensatory outcomes under different precommercial thinning regimes: 1 – over-compensation; 2 – exact compensation; and 3 – under-compensation.

Terms closely related to the term CG refer to quantities that are measurable at a given time, such as total biomass or merchantable volume of the disturbed stand compared with biomass or volume of an undisturbed stand. These comparisons could result in:

- *Under-compensation*, in which accelerated growth in the disturbed stand has not yet yielded biomass equal to that of the control (undisturbed) stand;
- *Compensatory-induced-equality* (CIE, or exact compensation), in which the accelerated growth in the disturbed stand has allowed its biomass to become equal to that of the control stand;
- *Over-compensation*, in which the biomass of the disturbed stand has exceeded that of the control stand;

These three conditions refer to particular points in the growth cycle of a species as illustrated in Figure 1. In slow growing trees, the categorization may also change with time, as with the upper curve in Figure 1. Numerical examples of CG in forestry were presented in Li et al. (2020).

These terms might require somewhat different definitions in different situations. For example, in insect populations, the terms under-compensation, exact compensation, and over-compensation have been used for populations that reproduce once each year and in which the final population size before diapause may be below, at, or above the mean population size at carrying capacity (Varley et al., 1973). If the life cycle is such that adults die after reproduction, then the compensatory response and population level may oscillate back and forth across the mean population size for some time if the response is over-compensatory. If response is over-compensatory, then the terms and allocations applied to growth become identical to the compensation terms applied to population sizes (or volumes) at the end of the life cycle. For trees, with their extended life cycles, the growth terminology and volume terminology need to be separated. For trees, the status of volume can change from under-compensation to exact (or CIE), and later to over-compensation, so that the age and status of the tree's growth cycle must also be specified.

A further complicating issue for trees is the age structure of the population. If some disturbance kills a significant proportion of an uneven-aged natural stand, then ingrowth of young trees will occur or be accelerated, although the effects of this on stand volume may or may not be evident for some time. This may be especially evident if the disturbance is caused by insects that kill mainly one size class of trees, for example from damage by the mountain pine beetle (Safranyik, 2004). On the other hand, if the stand is planted and managed, the stand will tend to be even-aged, and the response may be more predictable; the same response of ingrowth may occur, but the loss by the disturbance may produce a response largely consisting of increased somatic growth of individuals.

### Objectives of This Review and Synthesis

The objectives of this review are 2-fold. One is to provide a comprehensive review of diverse concepts and examples of CG studies from different research fields to show the ubiquity of CG phenomenon, and the other is to describe existing understanding, modeling and applications of CG and how the lens of CG could benefit forestry research and practice.

In the sections Compensatory Growth in Trees and Analogous Compensatory Growth in Other Plants and Animals, we provide evidence for diverse CG in forest trees and a wide range of organisms. We show that the phenomenon is not simple but can be understood when the appropriate framework is applied. In the section Understanding and Predicting Compensatory Growth, we summarize understanding of CG and associated modeling efforts; and in section Discussion, we discuss the analogs in CG research and their related scaling-up issue, as well as the usefulness of lens of CG. This article will end with some concluding remarks and recommendations for future research.

## Compensatory Growth in Trees

### Compensatory Growth in Hardwood Tree Species

Morphological responses have been observed after experiencing a disturbance. Rea and Massicotte (2010) reported that the sizes of shoots, leaves, and other plant parts of hardwood species after cutting, animal browsing, or breakage from snow could typically fall outside of the normal ranges given in plant identification keys. The authors provided five examples collected in or near Prince George, British Columbia, Canada, to illustrate these abnormal situations including:

1) Increased growth of leaves of dogwood (*Cornus stolonifera Michx*.) following heavy winter browsing by moose;
2) Leaves of *Viburnum opulus* L. following cutting damage;
3) Large leaf production on a compensatory branch of white poplar (*Populus alba* L.) after damage from an ice storm;
4) A regenerating annual shoot produces large leaves on elm (*Ulmus americana* L.) in the year after autumn pruning;
5) A large compensatory leaf from a shoot growing from a cottonwood (*Populus balsamifera* L.) tree stump after clearcut logging.

Rea and Massicotte (2010) successfully employed the lens of CG to explain these observations, and encouraged students to think “outside the box” to understand why growth varies in some plant parts and reinforces the value of dichotomous keys.

As a major type of animal-plant herbivory relationship, animal browsing can also cause changes in tree biomass. For example, Guillet and Bergström (2006) studied the effect of timing and intensity of deer browsing on compensatory response of fast-growing willow (*Salix*) grown for bioenergy. In their study, browsing was simulated several times during summer and once during winter. The clipping was done at different intensities during the first year after establishment in a new willow plantation, and during the first year after harvest in an older willow coppice. The authors recorded the total aboveground biomass and biomass available for deer browsing both at the end of the first and the second growing periods after clipping. They found that in both the new plantation and in the older coppice, the willows fully compensated for biomass losses after winter clipping, irrespective of clipping intensity. In the older coppice, moderate clipping intensities, corresponding to usual browsing levels by deer in summer, did not influence total produced biomass. Guillet and Bergström observed that the newly established willow stools reacted to summer clipping by undercompensating in terms of biomass available for deer browsing, while older stools could overcompensate. Compared to stools clipped in late summer, willow stools clipped during early summer were able to compensate earlier and were stronger. They thus concluded that depending on the circumstances, the fast-growing willows responded within the whole continuum from under- to overcompensation.

Erbilgin et al. (2014) investigated the mechanisms used by plants to cope with the impact of herbivore damage. In a greenhouse experiment, they evaluated CG for trembling aspen (*Populus tremuloides*) seedlings under varying intensities and frequencies of simulated defoliation, with or without nutrient enriched media. They found that “all defoliated seedlings showed biomass accumulation with low defoliation intensity and frequency, regardless of resource availability; however, as defoliation intensity and frequency increased, CG of seedlings was altered depending on resource availability. Seedlings in a resource-rich environment showed complete compensation, in contrast responses ranged from undercompensation to complete compensation in a resource-limited environment.” Instead of plant resistance mechanisms (i.e., any plant trait that prevents or reduces the amount of herbivore damage), Erbilgin et al. (2014) treated the CG as a plant tolerance mechanism (i.e., the degree to which plant fitness is altered following damage), which is common in woody plants including deciduous and coniferous trees.

### Compensatory Growth of Balsam Fir

The growth responses of balsam fir (*Abies balsamea* (L.) Mill.) to PCT have been reported differently in short- vs. long-term observations. For example, Schneider et al. (2013) studied a 28-year PCT effect on balsam fir at the Réserve faunique de Matane, ~400 km to the northeast of Quebec City, and found that the treatments did not significantly increase stand yield, nor change stand diameter diversity or distribution.

Pitt and Lanteigne (2008) reported that the stand volumes in treatment sites were about 15% higher than those from untreated sites, 42 years after the initial PCT treatments in the Green River watershed of northwestern New Brunswick, ~48 km north of the town of Edmundston. Their analysis was based on the estimates of cumulative stem volume losses through mortality in non-thinned stands and the gains from individual stem volume, calculated from the increase of quadratic mean diameters. In his establishment report for the Green River thinning trials, Baskerville (1959) forecasted that “with cleaning and thinning, it should be possible to produce 30 cords per acre in 40 years.” Pitt and Lanteigne (2008) concluded that 40 years later, that his forecast fell short by 2-fold, and growing confidence was gained that there is room for silviculture to enhance the productivity and competitiveness of the northern forests.

To validate the predictions of studwood volume, sawlogs, and pulpwood of the full Green River dataset, three of the six replications were clearcut in the fall of 2008. The results indicated that thinning to a nominal spacing of 6 ft led to 1,600 merchantable stems per ha by stand age 30, which offered the best balance of individual tree and stand growth. This produced 20% more gross merchantable volume and 26% more sawlog volume than non-thinned stands, potentially increasing landowner stumpage revenues by 22% (Pitt et al., 2013).

In addition to the different lengths of observation periods, the two case studies had different thinning intensities, i.e., much higher thinning intensities were applied to the Réserve faunique de Matane case (thinned to 625–750 stems per ha) than that in the Green River case (thinned to 6,267–1,682 stems per ha). Therefore, direct comparison of the results from these two case studies can hardly be generalized to the general evaluation of PCT effect.

### Compensatory Growth of Costal Douglas-Fir

Costal Douglas-fir (*Pseudotsuga menziesii* [Mirb.] Franco) has long been a focal species in silvicultural research such as the effect of PCT in North America and Europe. At the individual tree level, results are consistent on the released growth of crop trees after different thinning regimes, i.e., faster growth of diameter in thinned stands than that from unthinned stands. However, the results at the population/stand level showed diverse patterns of CG, ranging from positive to negative growth responses.

First, there are a number of studies that showed that the CIE (Li et al., 2020) was reached after PCT treatments. The Schenstrom Thinning Plots (Schenstrom, 1931) provided perhaps the first opportunity to compare the development of a natural with a managed second-growth stand, established in the coastal region of British Columbia. There have been 12 measurements and 7 major thinnings for the plots. At the fiftieth anniversary of plot establishment, Warrack (1979) published the results of “taking into account site differences, figures indicate that whatever the treatment, including the unthinned control, there is no difference in growth volumes.”

A similar conclusion was achieved in a European study. Analyzing the data from Swiss Central Plateau, Schütz et al. (2015) studied the long-term effects of thinning regimes on stand development, and found that different thinning regimes do not have much impact on stand productivity 41 years after treatments. Similar observations were also obtained from coastal Douglas-fir forests in the Malcolm Knapp Research Forest of UBC in Maple Ridge, British Columbia, where more or less the same forest productivity was observed about 50 years after different thinning regimes (Cheryl Power, Pers. Comm., Dec. 2017). Somewhat similar forest conditions have also been observed for aspen mixedwood forests near Calling Lake, Alberta 40–50 years after various silvicultural treatments (Derek Sidders, Canadian Wood Fiber Centre, Pers. Comm., Nov. 2017).

Second, there are cases where overcompensation was observed sometime after PCT. For example, Steele (1955) reported 20 years results of two young Douglas-fir stands 20 years after PCT on the Wind River Experimental Forest in Skamania County, Washington. He found that thinning had not affected total cubic volume at the end of the period, though the volumes from thinned stand exceeded that from the control stand. However, thinning had apparently accelerated diameter and height growth, and substantially increased the board-foot volume now available in trees 7 inches in diameter and larger.

Li et al. (2018) reported that in a long-term coastal Douglas-fir silvicultural trial (combined PCT and fertilization) near Shawnigan Lake, Vancouver Island, British Columbia, Canada, the total stand volumes in most treatment sites exceeded that from untreated sites 40 years after initial treatments at both the tree and stand levels. The conclusion was based on data collected in multiple measurements of stand volumes, calculated by DBH and height of every surviving tree within the plots. The authors found that the length of period required for thinned stands to reach the CIE is dependent on the level of fertilization, and a quantitative relationship was then presented as a function of thinning intensity and fertilizer application.

Third, there are also cases showing a negative effect of CG. For example, Harrington and Reukema (1983) reported analytical results 25 years after 6 PCT treatments to a 27 years old Douglas-fir plantation on the Wind River Experimental Forest near Carson, Washington. They showed that a thinning shock happened immediately after the thinning, in terms of substantially reduction of height growth. Nevertheless, despite an initial negative response to PCT treatments, the CG proceeded after this initial thinning shock period.

Diverse patterns of CG reported for coastal Douglas-fir suggest that tree responses to thinning differed in different geographical regions, site conditions, and timing and intensity of thinning operations, etc. and it might be difficult to generalize the role of PCT on stand productivity. Results from this evaluation will also depend on the length of observation period, i.e., short- vs. long-term results might not be the same. Therefore, optimal thinning regimes may not always be equally applied to different forest stands, i.e., it's unlikely one solution will fit all.

## Analogous Compensatory Growth in Other Plants and Animals

### Compensatory Growth in Other Plants

The earliest report of CG in plant sciences can be tracked back to the 1920s. It was found as early as 1926 in Arizona, that cotton (*Gossypium hirsutum*) yields might be increased by artificially delaying, for several weeks, the normal initiation of boll setting (Eaton, 1927). Eaton observed that during the 36-day interval between the first and second defruiting, the 25 control plants increased the number of bolls carried at an average rate of 3.5 bolls per day; whereas the defruited plants increased the number of bolls carried at the rate of 10.7 bolls per day. This result was consistent with what observed in tomato (Murneek, 1926) in which plants with defruiting treatment carried a heavier boll load soon after defruiting than did untreated control plants. The observation provided a premise that an increased vegetative growth (floral-bud formation) and a decreased shedding rate followed when all previously set bolls were removed from cotton plants. These are perhaps the earliest examples of CG observed in annual plants of crops and vegetables, though the observation was not termed so at the time. The findings are also consistent with the common knowledge of florists and horticulturists that when only a few flowers or fruits on a plant are left to develop, a superior product may be expected. Based on all of these, “it seemed logical to assume that an increased yield would result if the first bolls set by cotton plats were sacrificed in favor of a larger plant with a greater initial photosynthetic capacity” (Eaton, 1931).

This phenomenon was generally termed *yield response* in the agriculture literature, describing how cotton yield could respond to the removal of the first set of bolls. Use of the term, CG, for describing this phenomenon did not occur until considerable efforts were devoted to explain the mechanisms of this phenomenon in 1980s (e.g., Crawley, 1983, in which plant compensation was described to occur at both the population and individual levels). At the plant level, a wide range of defense mechanisms and a variable capacity for CG could be documented through their response to herbivory or other disturbances. Populations could compensate when competition was relaxed by herbivore attack on one individual thus allowing another individual to grow more rapidly (e.g., Herms and Mattson, 1992). As pointed out by Hjalten et al. (1993) consequently, under-, equi-, and over-compensation may correspond to plant growth or yield becoming less than, equal to, or greater than undamaged controls. Brook et al. (1992) and Sadras (1995) demonstrated diverse yield responses of insect-damaged cotton crops spanning the whole range from over-growth to a net growth reduction.

Sadras and Henggeler (1994) defined compensation as the ability of the plant or crop to offset damage caused by pests or other factors such as hail. As shown in Crawley (1983), such compensation can act at the plant or crop levels. Plant compensation refers to the ability of individual plants to regrow after damage. Crop compensation may arise when damage is not uniform, i.e., crops compensate when insect attack on one individual plant allows its undamaged neighbor to grow faster. Sadras and Henggeler (1994) presented results of an experiment that showed that a plant adjacent to a damaged plant yields more than it would otherwise.

Haile et al. (1998) studied the response of soybean (*Glycine max* (L.) Merr.) cultivars to insect defoliation, and showed diverse pattern of CG. They found that weather conditions can play a role in determining insect tolerance and compensation of soybean. Their field experiments showed that the yield of three indeterminate soybean cultivars: Dunbar, Corsica, and Clark was not affected by defoliation in 1994 in which ample rainfall occurred during the soybean growing period, thus all cultivars compensated for all levels of defoliation via delayed leaf senescence and compensatory regrowth, which enhanced the light interception capacity of defoliated soybean canopies. However, in 1995, defoliation caused a significant yield reduction in all cultivars; this yield reduction was greater for Dunbar than the other two cultivars. In both years, yields were directly related to the light interception capacity of soybean canopies after defoliation.

Herbivory is generally referred to as animals eating plants. Grazing (mainly forage grass) and browsing (primarily consumption of tree leaves and/or twigs) are two main feeding strategies in herbivory. An intermediate feeding strategy is mixed-feeding. Here we focus on grazing feeding strategy that is closely related to practical rangeland management.

CG can be seen as a kind of plant reaction to herbivory and adaptation strategy. In a comprehensive ecological study in the Serengeti National Park, Tanzania, and Masai Mara Game Reserve, Kenya, McNaughton (1979, 1983, 1985) measured basic state variables of aboveground plant biomass inside permanent and temporary fences (control), and outside fences (with grazing by ungulates). He found that CG in plants subjected to herbivory may alleviate the potential deleterious effects of tissue damage, on either vegetative or reproductive organs. Tissue destruction is rarely translated into a proportional reduction of final yield. Based on these observations and the statistical relationships between plant productivity and grazing, he tested a hypothesis that grazing can increase grassland productivity and showed that aboveground productivity due to grazing was maximal at intermediate grazing intensities for mid-slope and flatland grasslands. The results suggested that CG mechanisms might be the driving forces determining plant productivity.

**Figure 2:**
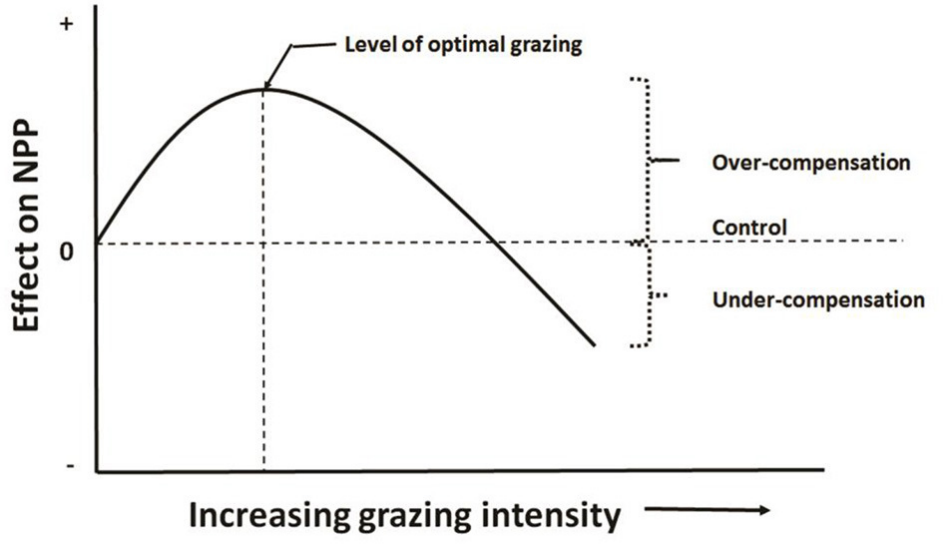
Herbivore-optimization curve (redrawn from McNaughton, 1983) showing level of optimal grazing occurs in mid grazing intensity, which can be defined as number of herbivores, their biomass, or the amount of tissue removed.

This hypothesis has been called grazing optimization hypothesis (Hilbert et al., 1981), or herbivore grazing optimization curve in Williamson et al. (1989), or herbivore-optimization curve in Belsky (1986) as showed in Figure 2.

Animals benefit plants by pollinating flowers, dispersing seeds, fertilizing soil with dung, and reducing the size of competing plants. However, there has been a debate on whether herbivory can increase the productivity, longevity, or reproductive potential of some plant species and whether such increases have resulted in an increased fitness of grazed populations and the coevolution of mutualisms between consumers and plants.

Belsky (1986) found that the ambiguity of the term CG has been a major confounding factor in the debate. CG, commonly defined as a positive response of plants to injury, has been used to describe plant responses ranging from a partial replacement of lost tissue to a net productivity exceeding that of uninjured control plants. To avoid such confusion, Belsky subdivided the one term into three: (1) *overcompensation* occurs when the cumulative total dry weight (including removed tissue) of the grazed or clipped plants is greater than the total dry weight of the control plants; (2) *exact compensation* occurs when the cumulative dry weight of the treated plants equals that of the controls; and (3) *undercompensation* occurs when the cumulative dry weight of the treated plants is less than that of the controls. Partial compensation takes place when undercompensating species produce more biomass than would be expected if no increase in growth rate occurred; they may make no compensation for the lost tissue; or the treatment causes damage (Crawley, 1983) such that the remaining tissue produces less than would be expected.

Williamson et al. (1989) experimentally tested the herbivore grazing optimization hypothesis, using grazing controlled densities (0 to 145 individuals/m^2^) of big-headed grasshoppers (*Aulocara elliotti* Thomas) for short time spans (7 to 13 days) on enclosed swards (0.7 m^2^) of blue grama [(*Bouteloua gracilis* (Willd. ex H.B.K.) Lag. ex Griffiths] in wooden frame cages. They measured aboveground net primary productivity (ANPP) of each of 257 experimental enclosures following regrowth by using a standing crop index (the product of mean total blade length per tiller and percent basal cover) after the grazing period and clipping after the regrowth period. Their results showed that ANPP was not significantly reduced by grazing in any of the 5 short-duration grazing experiments. In 2 of the 5 experiments, ANPP increased significantly with grazing. In 1 of the other 3 experiments there was evidence to support the grazing optimization hypothesis.

### Compensatory Growth in Animals

Observations of CG in animals can also be traced back to more than a century ago. Osborne and Mendel (1915, 1916) observed in their study of relationship between Vitamin A and growth using albino rats, *Mus norvegivus albinus*. They found that “certain mixtures of isolated food substances furnishing a ration upon which animals decline or cease to grow can be converted by the addition of some of the natural “fats” into a ration adequate for growth,” and animals would resume normal growth rate for the size at the time, after a period of unfavorable conditions. “It need not be slow, and frequently it actually exceeds the normal progress.” Brody and Elting (1926) demonstrated that growth, at least in the last stages, proceeds as if the normal condition were the mature size, and that the rate of growth is proportional to the growth needed to reach mature size. Consequently, an animal whose growth has been retarded exhibits, when the restriction is removed, a rate of growth greater than that of normal in animals of the same chronological age. Bohman (1955) has termed this abnormally rapid growth, relative to *age compensation*.

CG is widely observed in livestock animals at the individual level, and the farm animal production industry has benefitted greatly through applications of CG research results. In a review article, Wilson and Osbourn (1960) referred CG as to a phenomenon of rapid growth exhibited by mammals and birds after a period of growth restriction. The capacity of CG of manifesting the growing animals reared on restricted feeding or on the diets of low nutrient content may be advantageously utilized in livestock farming under the situation compelling the animals to feed at low plane of nutrition in the growing phase.

Carefully designed experiments are the primary method of studying CG at the individual level of animals. For example, Bohman (1955) published a 9-year study using 137 wintering weanling and yearling beef cattle fed with native mountain meadow hay harvested at two stages of maturity. He found that significantly greater gains were obtained by the cattle fed on early-cut hay during the winter feeding period. “The restricted animals gained significantly more during the following summer, and at the end of the first year, the cattle fed the better quality forage still weighed significantly more than the animals fed late-cut hay, but the difference was small. Animals kept during a second year completely compensated for the two winters of restricted growth and were as heavy by the second fall as the animals fed early-cut hay.” He then concluded that “animals restricted in growth were not permanently stunted and tended to compensate for this restricted period quite rapidly.”

In broiler chickens, early life with fast growth rate is often associated with broilers fed *ad libitum*, resulting in high body fat deposition, and a high incidence of skeletal and metabolic disorders. To solve these problems, increased interest has been attributed to the concept of CG. Zubair and Leeson (1996) reviewed CG in broiler chickens subjected to early life undernutrition. They found that factors influencing CG in the broiler chicken include “the nature, severity and duration of undernutrition, as well as the age at the commencement of undernutrition and the degree and pattern of re-alimentation.” Many studies showed improvement in feed efficiency during CG in restricted-refed broilers. Broiler chickens undergoing CG also exhibit greater than normal feed intake relative to body weight and some associated digestive adaptation. They then suggested that the use of this concept to address problems associated with early life fast growth rate requires more studies of the nutrition of the broiler chicken during the period of growth compensation.

Yambayamba and Price (1991) defined *catch-up growth* (CG) as “the acceleration in growth that occurs when a period of growth inhibition ends and favorable conditions are restored. It is essentially a self-correcting response which restores the individual to its original growth curve.” They used 53 Hereford crossbred heifers in their study on the effects of CG on carcass composition. With an experiment utilizing 2 and 4 month feed-restriction treatments and a control group, they found that feed restriction for 2 months had no significant effect on the composition of the three-rib cut, but 4 months of feed restriction was associated with significantly lower and higher proportions of fat and bone, respectively, in the three-rib cut. At the final slaughter weight, no significant differences were found among treatments in the tissue proportions of the three-rib cut. This indicated that 2 or 4 months of feed restriction, starting at 6 months of age, has no permanent effect on a heifer's live weight or body composition. Therefore, they concluded that the CG is of practical use in the beef industry, e.g., “certain feedlots commonly buy underfed steers, knowing they will catch up rapidly and efficiently.”

CG has also been demonstrated in fishing and aquaculture. Ali et al. (2003) defined CG as a phase of accelerated growth when favorable conditions are restored after a period of growth depression, which is important to fisheries management because it reduces variance in size by causing growth trajectories to converge and offsets the effects of growth arrests. They found that CG existed in many fish species including Herring (*Clupea harengus*), Atlantic salmon (*Salmo salar*), rainbow trout (*Oncorhynchus mykiss*), Coho salmon (*O. kisutch*), Sockeye salmon (*O. nerka*), Arctic charr (*Salvelinus alpinus*), Channel catfish (*Ictalurus puntatus*), African catfish (*Heterobranchus longifilis*), Atlantic cod (*Gadus morhua*), three-spined stickleback (*Gasterosteus aculeatus*), Yellow perch (*Perca flavescens*), pike-perch (*Stizostedion lucioperca*), hybrid sunfish (*Lepomis cyanellus* x *L. macrochirus*), Winter flounder (*Pleuronectes americanus*), and Alaska yellowfin sole (*Pleuronectus asper*), etc. in both individually housed and grouped fish, typically after growth depression has been induced by complete or partial food deprivation.

All ranges of partial, full and over-compensation have been found in fish at the individual level. Examples of full compensation has been observed in rainbow trout, barramundi, *Lates calcarifer*, Arctic charr, gibel carp, *Carassius auratus gibelio*, Atlantic salmon; partial compensation has been observed in hybrid tilapia, Persian sturgeon, *Acipenser persicus*, Arctic charr; no compensation has been reported in common carp, *Cyprinus carpio* L. and great sturgeon, *Huso huso* L. Overcompensation was observed in channel catfish and hybrid sunfish.

Applications of these individual level results to the population level have benefitted the aquaculture industries through determining optimal feeding practice, which is needed since overfeeding could lead to higher production costs and water pollution, whereas underfeeding could lead to poor growth performance and poor economical gain. In tilapia farming, high feed cost in traditional practices has made it difficult to convert commercial feed into economic gains. Gabriel et al. (2018) found CG response of hybrid tilapia (*Oreochromis mossambicus*) subjected to short-term cycles of feed deprivation and refeeding could contribute to the reduction of feeding cost, as well as reducing water quality problems and labor cost. However, diverse patterns of CG in tilapia species have been reported such as complete compensation by Cuvin-Aralar et al. (2012) and Passinato et al. (2015); partial compensation by Wang et al. (2000) and Gabriel et al. (2018), and no compensation by Gao et al. (2015). These results were obtained under different experimental systems and feeding protocols. They provide evidence for aquaculture industries to develop their own feeding strategies.

Azodi et al. (2016) studied the effects of various starvation and refeeding periods on growth, feed utilization, and body composition in Asian sea bass (*Lates calcarifer*), an important aquaculture species in the Indo-Pacific region produced commercially in ponds, cages, and recirculating tanks in both fresh and seawater. At the end of the experiment, there were no significant differences in growth and feeding performance among different treatments. Daily feed intake was significantly higher in the deprived fish than in the control fish. Sea bass showed complete compensation indicating a high ability of the deprived fish to grow sufficiently to fully compensate for weight loss during starvation. These results suggested that the feeding schedule involving starvation-refeeding cycles could be a promising feed management option for the culture of this species.

In aquatic animals, CG has also been found in crustaceans, in which growth intermittently accompanied molting activities. The evidence for CG in crustaceans, however, tends to be equivocal. Bostworth and Wolters (1995) demonstrated CG in juvenile red swamp crawfish, *Procambarus clarkii* (Girard), and Wu et al. (2001) demonstrated that both complete and partial CG occur in Chinese shrimp, *Fenneropenaeus chinensis* Osbeck. However, Paul et al. (1994) failed to reveal growth compensation in two brachyuran crabs, *Chionoecetes bairdi* Rathbun and *Cancer magister* Dana.

## Understanding and Predicting Compensatory Growth

The foundation for predicting CG, especially overcompensation, requires a good understanding of it. Several basic theoretical questions need to be addressed for the potential enhancement of forest productivity, such as whether observed forest productivity represents its maximal. If the answer is negative, why trees not utilize resources in full? Does partial mortality in earlier development stage would negatively impact on later stand development? Does partial mortality of a stand would result in possible extra-elevated growth rates than usually predicted by the conventional growth equations of natural stands? Fortunately, considerable effort has been made to parallel questions in the field of behavioral ecology, in which insects are widely used in experiments due to their short lifespan. We provide examples of these studies to assist understanding CG and overcompensation in trees and forests.

### Understanding Overcompensation

A basic issue of exploring overcompensation is to determine the maximal growth rates that could allow for CG and overcompensation to occur. The observed growth level is often thought to be suppressed relative to potential. This appears common in biology. In insect ecology, for example, Tammaru et al. (2004) tested the idea that a maximizer should be unable to further improve its growth performance, using the geometrid moth *Epirrita autumnata*, a spring-feeding species, which has been suggested in literature to be strongly selected for high growth rates, and may be growing at its maximum physiological potential. However, they found the larvae responded to starvation treatments by a subsequent compensatory increase in their relative growth rates, and such a plasticity may indicate that the rates of gaining weight are not maximized. They concluded that endogenous regulation of growth rates is widespread, and it remains to be shown if true maximization ever exists in nature. They also showed that the possibilities to compensate for adverse conditions experienced in early in larval development appeared to be limited, indicated by the strong correlation between initial weight in the last instar, and pupal weight.

A following question asks why are growth rates typically submaximal? De Block and Stoks (2008) tested the hypothesized direct costs of CG in terms of oxidative stress using the larvae of the damselfly *Lestes viridis*. Since CG in the larval stage was associated with higher oxidative stress, they assessed oxidative stress in body mass by exposing larvae to a transient starvation period followed by *ad libitum* food. They found that age and mass at metamorphosis will not necessarily completely translate larval stress into adult fitness and that the observed physiological cost may explain hidden carry-over effects. Their study also supports the notion that costs of CG may be associated with what they called free-radical-mediated trade-offs and not necessarily with resource-mediated trade-offs. Therefore, there is potential for CG and overcompensation to readily occur.

When resource utilization is submaximal, does an optimal strategy of resource utilization exist in governing plants' resource acquisition for growth and reproduction (and animals' foraging and movement patterns) that could be favored by natural selection? A positive answer means that a fixed proportion of onsite resources will be utilized by trees for growth and reproduction, thus the observed growth patterns should be related to the soil fertility, which is exactly assumed in the site index-based conventional growth equations (e.g., King, 1966). A partial mortality in a stand generates elevated resource availability for each individual tree on average, which results in faster growth rates of surviving trees than that from a normal stand without such mortality, as widely reported in forestry literature (see section Compensatory Growth in Trees). Research results from behavioral ecology also indicated that an optimal strategy exists as patch leaving rules for animals living in a patchily distributed resource environment (Charnov, 1976; Wajnberg et al., 2000). Charnov (1976) defined it as the marginal value theorem that “the predator should leave the patch it is presently in when the marginal capture rate in the patch drops to the average capture rate for the habitat.” When environmental quality is reduced to a certain level, animals will likely depart for a new patch with better quality. This rule appeared universal even facing uncertainties such as for parasitoids with imperfect host discrimination (Rosenheim and Mangel, 1994) and involved with differences of individual experience (Li et al., 1997) or “personality” (Stamps and Groothuis, 2010; Tremmel and Müller, 2012). These results have been extended to light-foraging decisions in a plant species that exhibits rapid movement: *Mimosa pudica* (L.) (Simon et al., 2016) and human decisions (Hutchinson et al., 2008).

A subsequent question is whether partial mortality at an early stand development would negatively impact later stand development, or whether a potential of partial morality could lead to CIE or even overcompensation. A parallel question in insect behavioral ecology is, whether periods of poor nutrition during early development can have negative fitness consequences in subsequent periods of ontogeny/adult stage. Dmitriew and Rowe (2005) examined the effects of a temporary period of food restriction on subsequent growth and age and size at maturity in the larval damselfly *Ischnura verticalis*. They also investigated whether this temporary period of reduced nutrition affected subsequent foraging behavior under predation risk. They found that increased growth rates later in development ensured that adult body size measurements (head and pronotum widths) did not differ between the treatments upon emergence. However, adult dry mass did not catch up to that of the controls, indicating that the increased growth rates for size dimensions occur at the cost of similar gains in mass.

Dmitriew and Rowe (2011) further tested the environment-matching hypothesis, in which a plastic developmental response to poor nutrition results in an adult phenotype that is better adapted to restricted food conditions than one having developed in high food conditions. They assessed the effects of larval food conditions (low, improving and high food) for the ladybird beetle (*Harmonia axyridis*) on reproductive fitness in both low and high food adult environments. They found no evidence that food restriction in larval ladybird beetles produced adults that were better suited to continuing food stress. The reproductive rate was invariably lower in females that were reared at low food, regardless of whether adults were well fed or food stressed. Juveniles that encountered improving conditions during the larval stage compensated for delayed growth by accelerating subsequent growth, and thus showed no evidence of a reduced reproductive rate. However, these same individuals lost more mass during the period of starvation in adults, which indicates that accelerated growth results in an increased risk of starvation during subsequent periods of food stress. Dmitriew and Rowe's (2005, 2011) studies indicated that adult fitness may not be significantly influenced by the larval food quality, that suggested that the final stand productivity may not be that matter on whether a partial mortality is experienced in its early development, or a CIE might always be possible to reach as long as enough time is satisfied.

Since levels for nutrient such as nitrogen varies among different plants, an interesting question to pose is whether partial mortality of a stand would result in possible extra-elevated growth rates than usually predicted by the stand density management diagram (Drew and Flewelling, 1979). Reflected in insect ecology, this question is equivalent to whether the feeding strategy of insects facing unevenly distributed food quality: either consume more low-nitrogen-level plants or suffer decreased performance. Lavoie and Oberhauser (2004) investigated growth and development of the monarch butterfly (*Danaus plexippus* L.) under different qualities of host plants in terms of nitrogen content. They applied high and low nitrogen fertilizer treatments to common milkweed grown in a glasshouse as different quality of food for the larval butterfly. High nitrogen fertilization resulted in increased leaf nitrogen and plant height, but also higher disease and pest levels. They found that monarch larvae compensate for lower plant quality by consuming more tissue, indicated by higher relative consumption rates. Monarch performance, measured with development time and relative growth rate, was affected differently in different larval stadia, but larvae of all stadia fed leaves from the low nitrogen fertilization treatment weighed as much as or more than those fed high nitrogen leaves. However, when increased consumption is costly because of increased exposure to natural enemies or increased expenditure on consuming and processing food, low nitrogen host plants may result in decreased fitness, despite the monarchs' ability to compensate. This may imply that an extra-elevated growth rate of a disturbed stand could be expected from a partial mortality due to higher level of nutrients utilization/acquisition. This also suggested that there could be a potential of enhanced growth and reproduction when extra resources become available, such as fertilization or mobilization of the unutilized proportion of onsite resources.

The above analogs are examples of mechanistic studies of CG that can provide a conceptual linkage for predicting CG in forestry.

### Modeling Compensatory Growth

Modeling techniques have been used in studying CG: two types of research can be found in the literature. One approach is used to demonstrate certain mechanisms that might contribute to CG, while another is employed to predict CG observed in nature.

**Figure 3:**
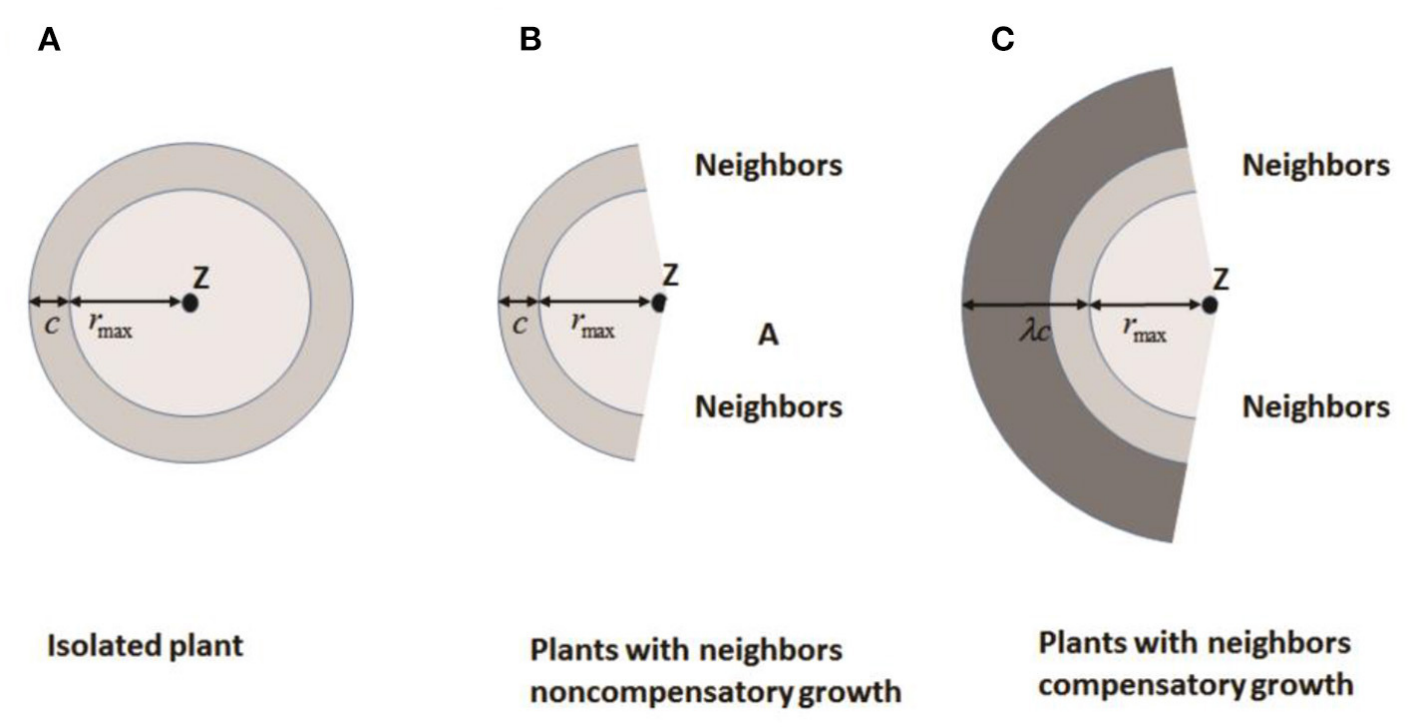
Increase in area of influence for: **(A)** an isolated plant; **(B)** a plant with no compensatory growth in the presence of neighbors, and **(C)** a plant with compensatory growth in the presence of neighbors. *Z* is the plant center, *c* is the radial increase of a circular plant in the absence of neighbors, *r*_*max*_ is the distance between a plant center and its most distant edge, and λ is a compensatory parameter (Redrawn from Brisson and Reynolds, 1997).

One example of the former regards root growth simulation models designed to demonstrate a possible mechanism that generates CG growth as below:

Bella (1971) defined the *area of influence* (AOI) as the circular area around a plant where it is effectively able to acquire resources. A “*neighborhood*” is generally defined as the circular area around a plant that contains all neighbors that affect its performance. The overlap in AOI of neighboring plants is used to estimate competitive pressure. Both AOI and neighborhood are based on a circle of fixed radius centered on a plant, which means that two plants in close proximity are always strong competitors. However, there is evidence that this is not always the case. Roots of many plants preferentially grow into areas of high resource concentration or into areas with less competition to compensate for close neighbors. Based on these findings, Brisson and Reynolds (1997) proposed a simple heuristic “compensatory” model of neighborhood interactions to explain such patterns. Assuming that the overlap of AOI between neighbors is minimal, the growth of individuals is symmetric (circular) until neighboring root systems meet (Figure 3A) where *c* is the radial increment of a circular plant in absence of a neighbor. When neighboring plants exist, the asymmetric growth of non-compensatory growth will stop at the edges of neighboring plants as shown in Figure 3B, representing loss from competition. For compensatory growth, asymmetric growth occurs in areas free of neighbors to compensate for the “loss of resources” in the zone of interaction by a single parameter λ as illustrated in Figure 3C. Therefore, non-compensatory growth is a special case of compensatory growth when λ = 1. With the increasing λ, the compensatory growth becomes stronger.

Thus, if space is available in its immediate vicinity, a plant may not necessarily be negatively affected by the presence of close neighbors. Their simulations show that compensatory plants are better able to utilize available space, have greater biomass, and outcompete non-compensatory plants. The change from a clumped to a regular distribution of individuals due to density-dependent mortality is delayed in non-compensatory plants. These theoretical results suggest that growth plasticity and the resulting asymmetry in space acquisition may play an important role in plant population dynamics.

Simulations that translate this utilization behavior of root systems as a mechanism of CG to population dynamics, showed the *rates* and *magnitudes* of changes in population attributes over time and on the competitive ability of individuals. The high rates of mortality in the population of non-compensatory plants is due to local crowding, despite the fact that a large proportion of the overall plot is unoccupied. Mortality in early stages of growth is delayed in plants with CG because of their ability to access available space outside their ecological neighborhood as defined by a circle with a fixed radius. That meant the compensatory plants can more efficiently utilize available resources to facilitate growth than non-compensatory plants. In the authors' simulation results, compensatory plants appear to utilize the available space better, generally occupying ~80% of the simulated plot, while non-compensatory populations never occupy >60% of the total area. This difference stems from both fewer individuals and the smaller average size of non-compensatory plants.

### Prediction of Compensatory Growth

There are two main approaches employed to model CG: statistical and mechanistic.

*Statistical modeling*: this descriptive approach is primarily used to fit equations to empirical data. Three examples demonstrate this approach.

**Figure 4:**
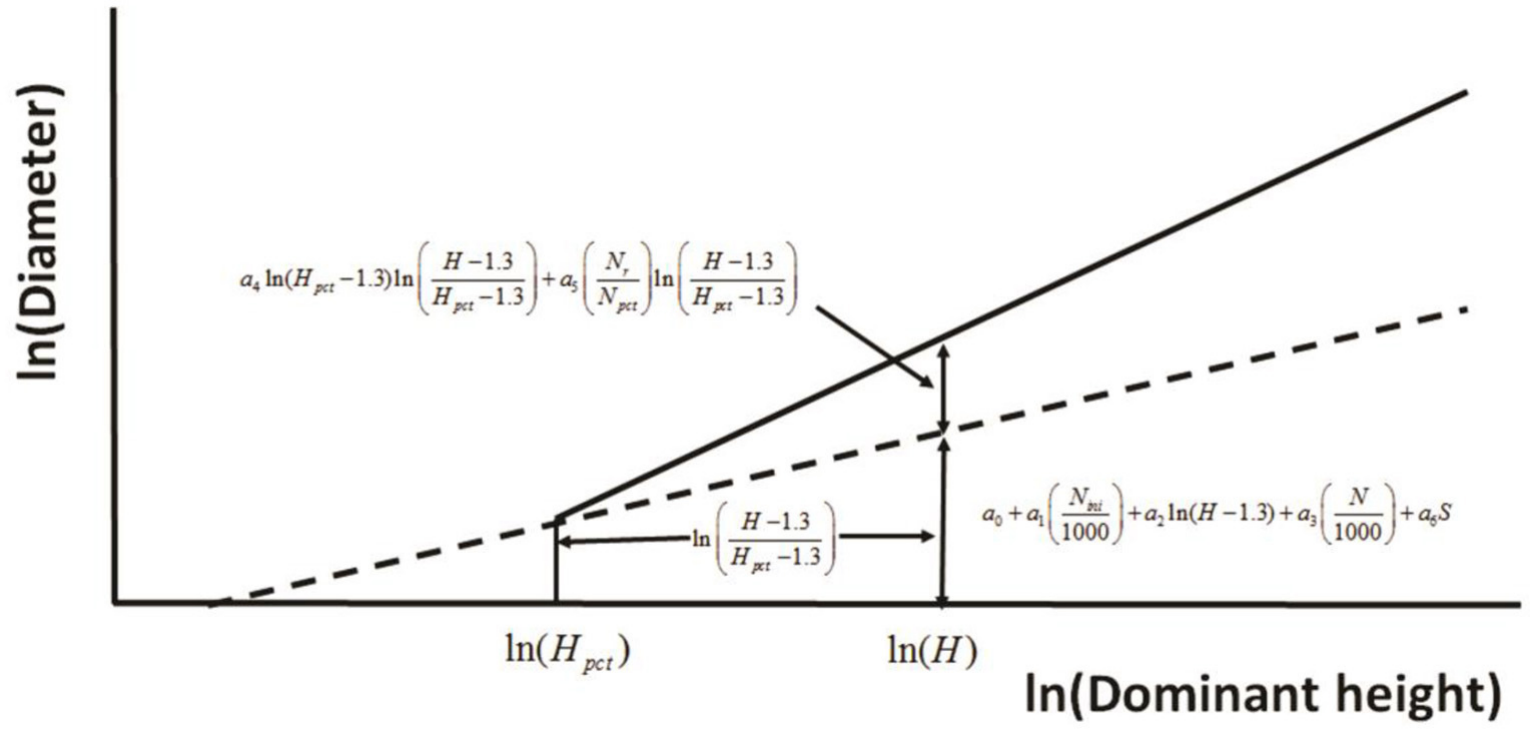
The principle used in defining the effect of precommercial thinning on the diameter development at a logarithmic scale (redrawn from Huuskonen and Hynynen, 2006).

Huuskonen and Hynynen (2006) developed a model to assess the response to thinning via stand diameter development of Scots pine (*Pinus sylvestris* L.) stands in Southern and Central Finland. The principle used in defining the effect of PCT on the diameter development at a logarithmic scale (Figure 4). In a stand where no PCT has been carried out, the diameter (*D*) development is predicted as a function of dominant height (*H*), initial stand density (*N*_*ini*_) and the actual stem number of the growing stock (*N*), and the regeneration method (*S*). If PCT is carried out, the predicted response in diameter increment is dependent on the timing of PCT (*H*_*pct*_, *H*–*H*_*pct*_,), and the thinning intensity, determined as the ratio between the number of removed trees (*N*_*r*_) and the stem number before thinning (*N*_*pct*_).

The final equation is thus expressed as:

(1) $$\begin{array}{l} \ln(D_{ik})=a_{0}+a_{1}\left(\frac{N_{ini,ik}}{1000}\right)+a_{2}\ln(H_{ik}-1.3)+a_{3}\left(\frac{N_{ik}}{1000}\right)\\ \;+PCT[a_{4}\ln(H_{pct,ik}-1.3)\left[\ln\left(\frac{H_{ik}-1.3}{H_{pct,ik}-1.3}\right)\right]\\ \;+a_{5}\left(\frac{N_{r,ik}}{N_{pct,ik}}\right)\left[\ln\left(\frac{H_{ik}-1.3}{H_{pct,ik}-1.3}\right)\right]]+a_{6}S_{k}+\mu_{k}+\varepsilon_{ik} \end{array}$$

where ln is the natural logarithm of the variable, and *a*_0_–*a*_6_ are parameters. PCT = 1 if PCT is carried out, otherwise it is 0. μ_*k*_ is the random stand effect and ε_*ik*_ is the random error. This relationship successfully represents short-term results because the data (169 plots in total from 13 experiments) used to fit the model were from years 20–29 of observation period and dominant tree height was up to 18 m.

The second example from animal CG research. Onoda et al. (2011) modeled the growth curve of body weight of male Thoroughbred horses of northern Japan using sigmoid sub-functions that incorporated seasonal CG caused by mating behavior. A non-linear mixed modeling method was employed to fit data of 1,633 body weights of the male horses to a weight-age relationship characterized by a general Richards growth curve equation showing a sigmoid curve.

(2) $$\begin{array}{l} BWT=\frac{575.0}{{\left(1.0+\left(-0.94513+0.3582f^{\prime }\left(t\right)\right)e^{-0.00213t}\right)}^{-\text{0}.8056719}} \end{array}$$

where *BWT* is the body weight, *e* is the base of the natural logarithm, and *f*′(*t*) is the sigmoid sub-function represents the seasonal adjustment factor:

(3) $$\begin{array}{l} f^{\prime }\left(t\right)=\frac{1.0}{1.0+e^{\frac{-10.0\left(t-432.0\right)}{268.49}}}-\frac{1.0}{1.0+e^{\frac{-5.0\left(t-432.0\right)}{268.49}}} \end{array}$$

where *t* is time (horse age in days). Their results showed improved goodness of fit to the weight-age data than that without using the sub-function, and was recommended for being used in estimating female body weight of Thoroughbreds or other growth traits affected by seasonal CG.

The third example employs the smoothing technology used for estimating the growth rates relative to control under different PCT intensities and years after PCT at the Shawnigan Lake study area by TableCurve 3D (Li et al., 2018):

(4) $$\begin{array}{l} z=a+bx+cy+dx^{2}+ey^{2}+fxy \end{array}$$

where *z* is the relative growth (stand volume in treated sites relative to that in untreated sites) in percentage, *x* is the PCT treatment represented by the volume removal in percentage, and *y* is the years after PCT. Therefore, the numbers of years required to reach CIE for different combinations of PCT and fertilization can be obtained by setting *z* to 100. This trend shows that a longer period would be required to reach CIE with increasing percentage of volume removal at the PCT, and the length of period required to reach CIE would be reduced with increasing utilization of fertilizer.

*Mechanistic modeling*: examples that demonstrate the effectiveness of this approach.

The first example is the model Shawn (Barclay and Hall, 1986), which is an ecosystem-based physiological model involving the effects of nutrients on tree growth, developed under the Shawnigan Lake Project. The model considered nitrogen movement in the soil, decomposition, mineralization, immobilization, nitrification, denitrification, external N inputs and uptake by tree roots, as well as the rates of incorporation of nitrogen from fertilizer present in the soil, both as organic and inorganic nitrogen. Individual trees were not identified, and only the biomass of various tree components were identified and incremented in the model. The model accommodated both thinning and fertilization treatments. A sensitivity analysis was performed that included 54 parameters and 36 variables to ascertain the importance of each of the parameters on the various processes and the resulting growth and mortality of the trees (i.e., biomass). Their results showed that the effects of parameters in the model largely mirrored the observed and measured effects of the treatments. For example, the Shawn model predicted that total wood volumes in thinned plots will be lower than that from unthinned plots right after the treatment, but will surpass that from unthinned plots after 50 years. This qualitative prediction has been confirmed by the data analysis of 40-year after initial treatments (Li et al., 2018). This example shows how the cumulative effect of PCT and fertilization can be significantly higher than that from the PCT treatment only.

The second example comes from the Tree and Stand Simulator (TASS) model (Mitchell, 1975), which predicted theoretically that stand basal area could be increased under PCT and/or fertilization. The TASS model simulates growth of trees based on the development of their canopy crown for managed stands, driven by species-specific site index curves (i.e., height over age curves). Crown increases in width and in length according to the height increment of the tree and it's position relative to other trees (e.g., crown contact with a neighboring tree stops crown expansion). The stem volume is expressed as a function of foliar volume, which is estimated from crown length and tree height. The stem volume distributes along the bole, and the bole increment increases linearly from the tree's apex to the base of the living crown. Below the living crown, the bole increment is equal to increment at the base of the crown. DBH or basal area growth is therefore a function of the bole increment model. The coefficients in the models that describe the processes above are calibrated using stand volume over top height from field data (e.g., experimental plots and permanent sample plots) to project tree growth (and hence, yield).

**Figure 5:**
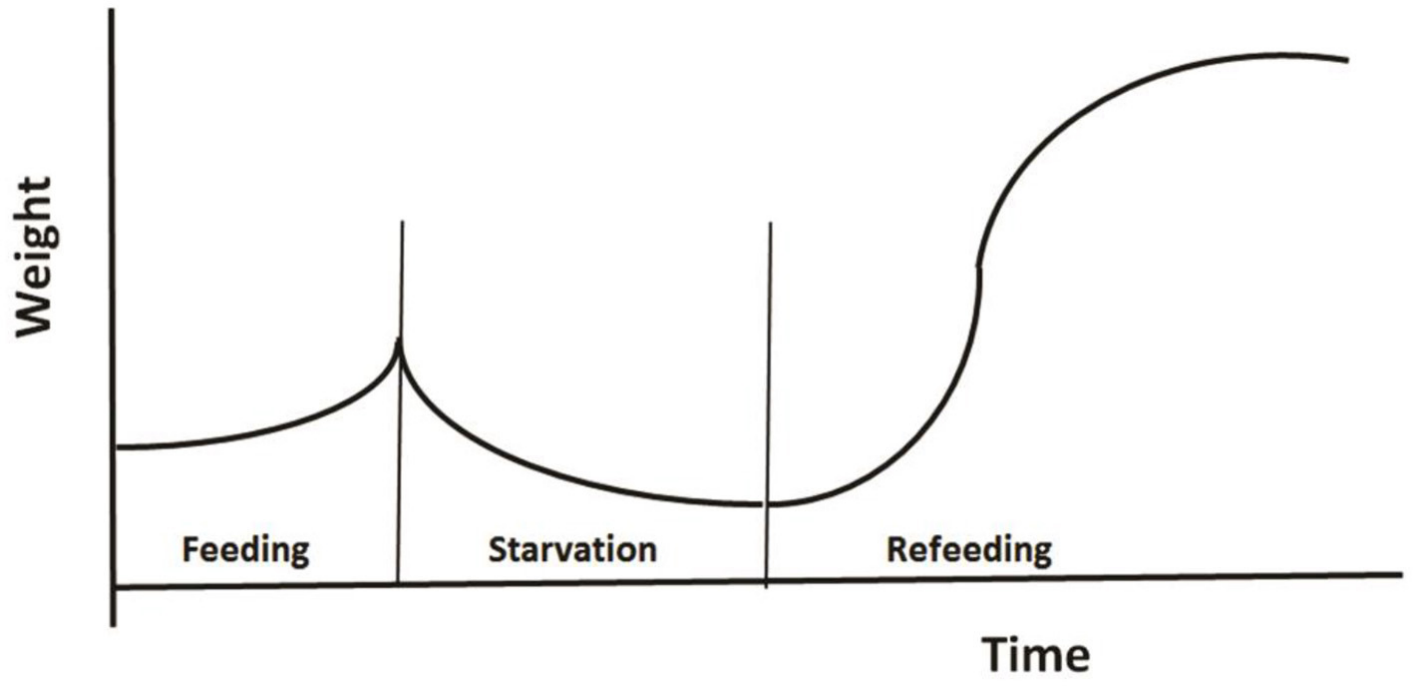
A typical pattern of weight change during a feeding-starvation-refeeding cycle, showing gradually increases under normal feeding and declines following food deprivation, then regains and may even reach a higher weight after refeeding (Redrawn from Broekhuizen et al., 1994).

The third example regards modeling the role of CG in changes of animal's weight as shown in Figure 5. Broekhuizen et al. (1994) developed a physiologically based model to investigate animal's growth pattern under different feeding regimes. Their model was based on two assumptions: (1) an individual partitions net assimilate between two tissue types: those which can and those which cannot be remobilized once laid down; and (2) the individual modulates its behavior and physiology in response to the instantaneous ratio of mobilizable to non-mobilizable tissues. The authors parameterized their model for salmonids using published studies of their energetics, and tested the model using the data from aquaculture studies of fish growth under conditions of fluctuating food. Using a common parameter set, the model successfully reproduces the growth patterns observed in 16 different feeding regimes.

## Discussion

### Analogs and Differences in Compensatory Growth Research

CG appears to be a ubiquitous phenomenon in biology. There is a large literature in the animal sciences that employ experimental methods that are suitable to determine the pattern of CG at the individual level. The experimental animals usually have a relatively short lifespan, and observations are made within a single generation. The most common metrics assess the growth responses in body weight after experiencing a period of deprivation or limitation of food, comparing with those from control groups. For annual plants, the most common treatment is damage of earlier reproductive organs and the response variable typically measures growth. Regarding plant CG in the face of herbivory, the treatments are usually the exposure of say, grassland to grazing by animals or feeding by insects. The results usually show that when food or nutrient supply is deprived, growth in body weight will stop, but will resume increasing when food or nutrient supply is restarted, and such increase is usually accelerated and results in catch-up growth, and in some case, the final body weight exceeds that of control group. This phenomenon is generally called CG, although it was not termed so when it was observed in early times.

An experimental approach is effective for short lifespan species because a researcher can design and implement many cycles of experiments during his or her professional career, but this approach might not be possible or valid for investigating CG in long-lifespan organisms such as trees. In addition, no experiment is perfect for covering the whole range of complexity in all possible conditions, thus it is difficult to determine the mechanism behind observed forest-based phenomena. Furthermore, it might also be difficult to determine the exact resource utilization strategy that trees used based on those imperfect experimental results and from there, scale up to industrial applications. Recognizing the above weakness of working with long-lived organisms, the strength may be also obvious, i.e., such organisms provide an unique condition of looking into CG in detail, because CG responses here can ensue slowly in real time.

Diverse CG patterns, even within a single species, as documented in the literature may simply result from different experimental designs and may be complicated by different experimental conditions, which are often difficult to control. Therefore, the results are not theoretically directly comparable without a formal meta-analysis. The very diverse results seemed confusing at the first glance—it is hard to determine if the phenomenon of CG exists and, if so, its exact pattern of its manifestation. Pitts (1986) pointed out that there is no single “fixed” pattern of CG, but there may be general principles that allow deepening understanding and predicting of the pattern, given the context. Searching for general CG principles appears to be particularly challenging. Mangel and Munch (2005) summarized that CG depends on species, social environment, Julian day, temperature, food availability, and physiological factors such as internal state and age.

Regarding CG in trees, the apparent similarity with animals is with the measurements made within a single generation for growth responses. In this regard, there is a distinct difference to the term “compensation” used in research of insect population ecology (e.g., Varley et al., 1973), in which the measurements are the reproductive success in between generations. Varley et al. (1973) used a new kind of reproductive curve, on logarithmic co-ordination, to show that different density dependent mortalities can have quite different effects on a population. Under-compensation occurs when the curve has a slope between 0 and 1, exact compensation will achieve when the curve has a slope of 1, and overcompensation can appear when the slope is larger than 1.

### Scale Issue in Practical Applications

Most studies of CG are conducted at the individual level, but practitioners may be interested in population level effects to benefit different industries. Here, scaling-up would involve transforming the results from individual effects to population consequences that may or may not be straightforward. For example, Picha et al. (2006) reported that tank-reared juvenile Hybrid striped bass (HSB), *Morone chrysops* × *Morone saxatilis* can undergo CG following limited food deprivation (maintenance feeding) and that the response is accompanied by improved feed efficiency. Toward industrial application of this knowledge, Turano et al. (2007) studied CG in pond-raised HSB, which is the typical form HSB aquacultures. They found that cyclic feeding elicited CG responses, and the feed efficiency was higher for all fish in the cyclic regimens. Their results suggested that the induction of CG through the practical application of different cyclic feeding regimens may show promise for cost savings and/or mitigating water quality problems for HSB producers. This kind of CG research has benefitted farm animal production and aquaculture industries significantly.

In agroecosystems, Eaton (1931) suggested increasing cotton yield at the level of crop or population through early removal of the reproductive organs of flower buds and squares would allow farmers benefit from the CG. Despite the benefits of CG at the individual cotton plant level, its application to the crop or population level in large area cotton fields may encounter significant challenges in terms of operational cost due to its labor-intensive nature. However, the challenges appeared relatively small in areas with lower labor cost such as China.

Cotton bollworm (*Heliothis armigera* (Hübner)) has generally 4 generations per year in North China, and the occurrence of the second generation is usually synchronized with the period when cotton flower buds and young squares appear. Traditionally, farmers made every effort to reduce the population density of cotton bollworm to protect as many flower buds and squares as possible because they are considered to be the key component of final cotton yield. These efforts resulted in a low population density of cotton bollworm as an economic threshold, and heavy application of chemical pesticide became almost a routine procedure that increased production costs and pesticide resistance significantly.

In the early 1980's, some insect ecologists studied this economic threshold intensively to improve farming economy. They examined yield response of cotton under different levels of cotton bollworm attack, during which they performed experiments of artificial removal of early flower buds and squares to examine the treatment effect on the final cotton yield. Different intensities of simulated larvae attack from cotton bollworm were carried out to determine the yield response. These studies allowed determination of economic threshold of the second generation cotton bollworm. The results indicated that, at the population (crop) level, CG can be used to increase cotton yield with reduced pesticide applications (e.g., Sheng, 1985). This example shows how farmers can benefit from CG research indirectly.

In summary, the benefit of CG research can be categorized into (1) improving farm economy through implementing optimal feeding strategy and reducing operational costs; (2) increasing final yield through overcompensation; and (3) reducing pesticide application in agroecosystems through elevating the level of the economic threshold.

### Usefulness and Applications of the Lens of Compensatory Growth

From a historical perspective, it would be hard to imagine that a population can survive changing conditions without responding accordingly. That is to say, organisms need to adapt or defend themselves to the changing environments, including the impact of climate change, to sustain their lives over the long-term. This means they must respond to the changes through altering behavioral patterns, and CG could be one of the options for this response. Consequently, most theory generally assumes CG is adaptive (Mangel and Munch, 2005). Following a period of deprivation, when resources become plentiful again, individuals may not respond at all and continue on a “normal” trajectory from a smaller size for their age, may exhibit faster-than-normal growth immediately following the end of the period, or may adopt a growth strategy that involves faster-than-normal growth at some later time. Compensating individuals may also overtake control individuals who have been growing normally throughout. It might be reasonable to assume that CG acts as an intrinsic capability of survival within a given species; however, whether it can reach the CIE or even overcompensation will depend on various internal and external conditions. CG, in fact, contributes to the resilience of forest ecosystems through absorbing some effects of negative disturbances. Mangel and Munch (2005) hypothesized that the key to understanding CG is that growth leads to the accumulation of damage at the cellular level that is expressed (and thus must be modeled) at the level of the organism. Therefore, some theories and research results from evolutionary biology and behavioral ecology could be borrowed to speed up CG research in forestry if such individual strategies can be scaled up to forest stands.

The framework of CG can provide a unified conceptual framework to explain diverse observations of forest growth patterns after disturbances with a partial mortality, ranging from under-, to CIE, and to over-compensation. A model based on this lens could serve as a basis for predicting dynamics of managed stands. It would be possible to have a baseline pattern (a state-dependent model) to represent CG and modified by individual internal and external states such as development ages/stages, the level of energy reserves, intensity of stimuli, speed of response, and period length of observation, etc. Such a model can assist practitioners to explicate the conditions that promote enhanced forest productivity through overcompensation in vegetative growth and thus benefit the forest sector as a whole (Li et al., 2020). The same principle can also be extended to include other natural disturbances that result in partial mortality of a forest stand.

Understanding CG can allow the prediction of forest responses to various disturbances, thus facilitate the implementation of active adaptive management that is suitable to meet challenges presented by climate change. Based on the concept of ecosystem resilience (Holling, 1973), adaptive management has been recognized as an effective measure of reaching given management goals through continuous improvement of existing management regimes (Walters, 2001). Active adaptive management is a proactive way of performing adaptive management; however, the risk of failure in experiments can be greater than that from passive adaptive management, which relies primarily on existing observations obtained usually under conservative and safe experimental designs. This is particularly true in silvicultural research.

Along with this approach, tool development is an essential need. We propose that a generic model should be developed first and then calibrated to given stand type and site conditions in specific geographic regions for CG predictions. The generic model needs to be state-dependent, i.e., separating lifespan of trees into a series of periods, within each period any disturbance including management operations-caused partial mortality, and tree's growth response to these actions accordingly (Li et al., 2020) could occur. Consequently, the growth trajectories of trees are the summation of growth responses in all these periods. The length of each period should reflect expected temporary resolution that satisfies the characterization of disturbances and desirable outcomes. To facilitate the expression of tree's “decisions” or response to partial mortality, an individual-based modeling approach (e.g., Korzukhina and Ter-Mikaelian, 1995; Ancelina et al., 2004) appears appropriate and scaling the results up to stand level through statistical summation. The details of model description is beyond the scope of current review, and will be presented elsewhere.

Collection of empirical datasets from legacy silviculture sites is also needed to calibrate and validate already- developed tools. Another research need is to explore and identify alternative indicators that allow sensitive detection of CG. Ideally, these alternative indicators should be closely related to forestry practice. When combined with forest evaluation methods, they can be useful in assisting operational decisions.

## Concluding Remarks and Recommendations

CG is common in many different plants and animals, and there is no theoretical basis to assume that trees are different, except being a much slower process to display because of their usual long lifespan. However, diverse patterns of CG sometimes confuse researchers on its existence. Expanding the range of CG patterns from negative to positive helped reach a consensus. Experimental methods in detecting CG are effective for short lifespan organisms, but not well-suited for trees, having a long lifespan. Even with the current incomplete understanding, CG research has benefitted different industries. To take full advantage of CG, the key is to understand possible mechanisms behind CG observations.

Studies of CG in forestry can speed translation of the CG concept and results from related fields. The direct advantage of such research is the possibility of enhancing forest productivity through overcompensation. However, diverse CG meanings need to be clarified to avoid misuse and/or misunderstanding. Development of a generic analytical model is recommended to predict possible patterns. With such an approach, not only mechanistic explanation can be achieved without waiting for long-term observations becoming available, but also different industries could benefit from the research results directly. This approach can also be efficient in pointing out future research direction or experimental design for maximized forest productivity.

## Data Availability Statement

The original contributions presented in the study are included in the article/supplementary material, further inquiries can be directed to the corresponding author/s.

## Author Contributions

CL, HB, BR, and RL jointly conceived and wrote the paper. All authors contributed to the article and approved the submitted version.

## Conflict of Interest

The authors declare that the research was conducted in the absence of any commercial or financial relationships that could be construed as a potential conflict of interest.
